# Fine-tuning anti-tumor immunotherapies via stochastic simulations

**DOI:** 10.1186/1471-2105-13-S4-S8

**Published:** 2012-03-28

**Authors:** Giulio Caravagna, Roberto Barbuti, Alberto d'Onofrio

**Affiliations:** 1 Institute for Informatics and Telematics, National Research Council, Via. G. Moruzzi 1, Pisa, 56127, Italy; 2 Dipartimento di Informatica, Università di Pisa, Largo Pontecorvo 3, Pisa, 56127, Italy; 3 Department of Experimental Oncology, European Institute of Oncology, Via Ripamonti 435, Milano, 20141, Italy

## Abstract

**Background:**

Anti-tumor therapies aim at reducing to zero the number of tumor cells in a host within their end or, at least, aim at leaving the patient with a sufficiently small number of tumor cells so that the residual tumor can be eradicated by the immune system. Besides severe side-effects, a key problem of such therapies is finding a suitable scheduling of their administration to the patients. In this paper we study the effect of varying therapy-related parameters on the final outcome of the interplay between a tumor and the immune system.

**Results:**

This work generalizes our previous study on hybrid models of such an interplay where interleukins are modeled as a continuous variable, and the tumor and the immune system as a discrete-state continuous-time stochastic process. The hybrid model we use is obtained by modifying the corresponding deterministic model, originally proposed by Kirschner and Panetta. We consider Adoptive Cellular Immunotherapies and Interleukin-based therapies, as well as their combination. By asymptotic and transitory analyses of the corresponding deterministic model we find conditions guaranteeing tumor eradication, and we tune the parameters of the hybrid model accordingly. We then perform stochastic simulations of the hybrid model under various therapeutic settings: constant, piece-wise constant or impulsive infusion and daily or weekly delivery schedules.

**Conclusions:**

Results suggest that, in some cases, the delivery schedule may deeply impact on the therapy-induced tumor eradication time. Indeed, our model suggests that Interleukin-based therapies may not be effective for every patient, and that the piece-wise constant is the most effective delivery to stimulate the immune-response. For Adoptive Cellular Immunotherapies a metronomic delivery seems more effective, as it happens for other anti-angiogenesis therapies and chemotherapies, and the impulsive delivery seems more effective than the piece-wise constant. The expected synergistic effects have been observed when the therapies are combined.

## Introduction

A key problem of anti-tumor therapies is finding a suitable scheduling of their administration to the patients. Of course a major problem in medical oncology is avoiding severe therapy-related side effects which, unfortunately, may cause the death of the patient. However, also in the ideal case of no side-effects, a therapy aims at reducing to zero the number of tumor cells in the host, within its end. Indeed, also if a single tumor cell remains the patient has a tumor. Actually, the requirement might theoretically be milder by accepting to leave the patient with a sufficiently small number of tumor cells so that the residual tumor can be eradicated by the immune system. In any case, both the duration and the scheduling of a therapy becomes of great relevance, as experimentally shown and theoretically studied [1]. In this paper we shall focus on a computational study of some kinds of immunotherapies, whose underlying key idea is to modify the natural interplay between tumor cells and immune system, by boosting the latter.

Tumor cells are characterized by a vast number of genetic and epigenetic events eventually leading to the appearance of specific tumor antigens, called neo-antigens. Such antigens trigger anti-tumor actions by the immune system [2], thus resulting in the tumor-immune system interaction taking place. These observations provided a theoretical basis to the hypothesis of immune surveillance, i.e. the immune system may act to control and, in some case, to eliminate tumors [3]. Only recently studies in molecular oncology and epidemiology accumulated evidences of this [4]. The competitive interaction between tumor cells and the immune system is extremely complex. As such, a neoplasm may very often escape from immune control. A dynamic equilibrium may also be established with the tumor surviving in a microscopic undetectable "dormant" steady state [5]. If this is the case, on the one hand a dormant tumor may induce metastases, on the other hand over a long period of time, a significant fraction of the average host life span, the neoplasm may develop strategies to circumvent the action of the immune system, thus restarting to grow [2,4,6,7]. We stress that this evolutionary adaptation, termed "immunoediting" [4], may negatively impact on the effectiveness of immunotherapies, as shown in [8].

Regarding immunotherapies, although the basic idea of immunotherapy is simple and promising [9], the clinical results are controversial since a huge inter-subjects variability is observed [10-12]. Immunotherapies are divided into two broad classes: passive and active therapies [13]. Among the passive ones, the *Adoptive Cellular Immunotherapy *(ACI) consisting in injecting cultured activated immune effectors in the diseased host [14,15] is probably the most important. Active immunotherapies aim at stimulating the immune response by expanding, for instance, the proliferation of cytotoxic T cells. Among these, a prominent role is played by *Interleukin-based therapies *[16,17].

Regarding the mathematical modeling of tumorimmune system interactions and and related therapies, many papers have appeared using various approaches. For instance, ordinary differential equations (ODEs) are used in [5,8,13-16,18-28], the theory of kinetic active particles is used by Bellomo and Forni in [29,30] and hybrid agent-based models have been introduced by Motta and Lollini [31,32]. In [14] Kirschner and Panetta proposed a largely influential ODE-based model of Tumor-Immune system (T-IS) interplay, whose variables are tumor cells, effector cells and the concentration of interleukins-2 (IL-2). This model is able to explain various kinds of experimentally observed tumor size oscillations [33-38] as well as both macroscopic and microscopic constant equilibria. Although a vast array of behaviors is mimicked by the solutions of the Kirschner-Panetta (KP) model, the tumor-free equilibrium is unstable for all biologically meaningful values of the parameters. However, in [39] we have shown that resetting the model in a hybrid setting where the interleukins are modeled with a continuous variable and the tumor and the immune system are modeled with discrete-state continuous-time stochastic process, the eradication via immune surveillance can be correctly reproduced. Since eradication is a fundamental topic in the study of immunotherapies, here we extend our hybrid version of the KP model to investigate the effects of both interleukin-based therapies and ACIs. Although our hybrid version of KP model is highly idealized, we think that it can provide useful information on the design of the above mentioned therapies.

## Methods

In the next sections we recall the KP model [14], its hybrid definition [39] and we extend the hybrid model with general immunotherapies.

### The deterministic Kirschner-Panetta model

In [14] the following model of the dynamics of tumor-immune system interaction was proposed

(1) $$\begin{array}{ll} {T^{\prime }}_{*}\; & =\;rT_{*}\left(1-bT_{*}\right)-\frac{p_{T}T_{*}}{g_{T}+T_{*}}E_{*}\;\\ {E^{\prime }}_{*} & =\frac{p_{E}I}{g_{E}+I}E_{*}-\mu_{E}\;E_{*}+cT_{*}+\sigma_{E}\;\\ I^{\prime }\; & =\;\frac{p_{I}T_{*}E_{*}}{g_{I}+T_{*}}-\mu_{I}I\;+\sigma_{I}\;\\ & \; \end{array}$$

where T_* _(*t*), E_* _(*t*) and *I *(*t*) denote, respectively, the densities of tumor cells, effectors of the immune system and interleukins. The tumor induces the recruitment of the effectors at a linear rate *cT *thus *c *may be seen as a measure of the immunogenicity of the tumor. In other words, according to [14]*c *is "a measure of how different the tumor is from self". The proliferation of effectors is stimulated by the interleukins. The average lifespan of effectors is $\mu_{E}^{-1}$ and the average degradation time for interleukin is $\mu_{I}^{-1}$. The source of interleukin is modeled as linearly depending on effectors, and it also depends on the tumor burden. Finally, continuous infusion immunotherapy may be delivered when effectors and interleukins are injected at constant rates *σ_E_*, *σ_I _*≥ 0.

In the case of no therapy, i.e. *σ_E_*, = *σ_I _*= 0, the main results obtained in [14] are that (*i*) the tumor-free equilibrium point (0, 0, 0) is always unstable, (*ii*) it exists positive *c_m _*≪ 1 such that for *c *∈ (0, *c_m_*) there is only one is locally stable equilibrium, whose size is very large due to the low value of *c*, (*iii*) it exists a *c_M _*> 0 such that if *c *∈ (*c_m_*, *c_M _*) there is a unique periodic solution whose period and amplitude decrease if *c *increases and, finally, (*iv*) when *c *>*c_M _*there is a unique globally stable equilibrium, whose size is a decreasing function of *c*. Thus, we note that this model explicitly precludes the possibility of tumor suppression in absence of immunotherapies. If idealized infinitely long constant continuous infusion therapies are considered the behavior of the system is complex, but in all the possible meaningful combinations of the parameters it is possible to find regions where globally stable limit cycles exist, as well as regions where there is cancer suppression. Moreover, there is a threshold value such that, for higher values of *σ_I_*, there is an unbounded growth of effectors, leading to severe side-effects.

### A hybrid model with constant therapies

As discussed in [39], the low-level oscillations predicted by model (1) make stochastic effects on the cell populations worth investigating. Unfortunately, it is possible to see that a purely stochastic model with discrete populations becomes computationally too hard to analyze, being the number of IL-2 huge. In this case a hybrid approach, despite being more costly than the deterministic counterpart, still permits a feasible analysis.

We now recall the hybrid model of T-IS interplay that we defined in [39] and that extends the deterministic model (1). Variables *T *and *E *of the hybrid model are obtained from the densities T_* _and E_* _in (1) converted into total number of cells by means of the volume *V *(e.g. the blood and bone marrow volumes for leukemia). We have T_* _= *TV *^-1 ^and E_* _= *EV *^-1^. This leads to the ODE system

(2) $$\begin{array}{c} T^{\prime }\;=\;rT\left(1-\frac{b}{V}T\right)-\frac{p_{T}T}{g_{T}V+T}E\\ E^{\prime }=\frac{p_{E}I}{g_{E}+I}E-\mu_{E}E+cT+V\sigma_{E}\\ I^{\prime }\;=\;\frac{p_{I}}{V}\frac{TE}{g_{I}V+T}-\mu_{I}I+\sigma_{I}. \end{array}$$

Note that the modified deterministic model (2) is obtained by the original Kirschener-Panetta deterministic model (1) by means of a nonsingular linear transformation. As it is very well-known linear transformations of the state variables do not change the topological properties of the solutions, and as a consequence these transformations do not change all the stability properties of equilibria [40,41].

From (2) a bi-dimensional stochastic process ruling the dynamics of *T *(*t*) and *E *(*t*) is linked to a scalar differential equation ruling the dynamics of *I *(*t*). In [39] it is discussed that the dynamics of *I *(*t*) can be assumed to be approximated by a linear ODE with randomly varying coefficients, which are constant in the intervals between two consecutive stochastic events. We briefly discuss how this model is simulated, for a detailed description of the underlying algorithm we refer to [39]. The algorithm adopted is an extension of the Gillespie *Stochastic Simulation Algorithm *(SSA) [42,43]. The SSA simulates a trajectory of the continuous-time discretstate Markov process underlying the system. To use this algorithm we write the stochastic events as reactions modeling birth and death, which for this model are described in Table 1. The set of all the events is denoted as R. At each step the SSA solves equations to discover the putative time for the next reaction to fire, and probabilistically decides which reaction fires. Intuitively, given $S=R\backslash \left\{R_{4}\right\}$ to each reaction $R_{i}\in S$ a propensity function *a_i _*(**x**) is associated. With the system state **x **at time *t *the value *a_i _*(**x**) *dt *gives the probability of the next reaction to fire in the infinitesimal time [*t*, *t *+ *dt*). All the propensity functions in S are time-independent, meaning that they depend on a state which is constant in between two stochastic events. Differently, *R*_4 _depends on the continuous part of the hybrid model, i.e. *a*_4 _(*t*) depends on *I *(*t*), and is hence time-dependent.

**Table 1:** Birth and death reactions for the hybrid model

reaction	Propensity	reaction	propensity
*R*_1 _: *T *↦ *T *+ 1	*a*_1 _= *r*_2_*T*	*R*_2 _: *T *↦ *T *- 1	$a_{2}=\frac{r_{2}b}{V}T^{2}$
*R*_3 _: *T *↦ *T *- 1	$a_{3}=\frac{p_{T}TE}{g_{T}V+T}$	*R*_4 _: *E *↦ *E *+ 1	$a_{4}\left(t\right)=\frac{p_{E}EI\left(t\right)}{g_{E}+I\left(t\right)}$
*R*_5 _: *E *↦ *E *- 1	*a*_5 _= *μ_E_E*	*R*_6 _: *E *↦ *E *+ 1	*a*_6 _= *cT*
*R*_7 _: *E *↦ *E *+ 1	*a*_7 _= *Vσ_E_*		

Reaction-based events and propensity functions [42] of the hybrid model [39].

We remark that the original SSA is assumed to simulate only time-independent chemical reactions. The algorithm used to simulate this model takes inspiration from algorithms simulating hybrid systems with time-independent propensity functions [44,45] and algorithms simulating purely stochastic systems with time-dependent propensity functions [46,47]. In [39] the original SSA equations (i.e. see [43]) for the putative time for the next reaction are rephrased in this setting. In particular, with the last stochastic event fired at time *t_n _*the putative time *τ *for the next reaction to fire is determined by solving

(3) $$\tau \underset{j\in S}{\sum }a_{j}\left(x\right)+\int_{t_{n}}^{t_{n}+\tau }a_{4}\left(t\right)dt=\chi$$

with *χ *a random number with distribution *Exp*(1). The solution of equation (3) has no analytical form, thus requiring iterative methods to find its solution.

We remark that the same equation in the case of only time-independent propensity functions yields the well known SSA strategy to generate exponential jumps. As far as model analysis is concerned, differently from the deterministic model (1), the stochastic simulations performed in [39] show that, at least in some cases, suppression of the neoplasm might be reached, thanks to the conjunction of the intrinsic tendency of the Tumor-Immune System to oscillate with the stochastic dynamics.

### A hybrid model with general therapies

In this paper we consider more general immunotherapies than the constant ones considered in [14,39]. Thus, here we allow that the therapy related influxes *σ_E _*and *σ_I _*are functions of time, and in next sections we shall focus on two common periodic scheduling of a therapy. In the deterministic setting, model (2) can trivially be modified by adding time-dependent immunotherapies, that is

(4) $$\begin{array}{c} T^{\prime }=rT\left(1-\frac{b}{V}T\right)-\frac{p_{T}T}{g_{T}V+T}E\\ E^{\prime }=\frac{p_{E}I}{g_{E}+I}E-\mu_{E}E+cT+V\sigma_{E}\left(t\right)\\ I^{\prime }\;=\;\frac{p_{I}}{V}\frac{TE}{g_{I}V+T}-\mu_{I}I+\sigma_{I}\left(t\right). \end{array}$$

It is important to notice that in the realistic case of finite duration therapies the deterministic system always predicts tumor re-growth, being the tumor-free equilibrium (0, 0, 0) unstable. Practically, if at the end of the therapy the solution is very close to the tumor-free equilibrium, immediately after the end the tumor restarts growing. We remark that in the oncological context it is important the state in which the tumor is at the end of a therapy, e.g. the tumor shrinkage up to an undetectable size, but it is far more important what is observed during the follow-up visits, i.e. that the tumor has not re-grown. In this framework the deterministic model is unable to reproduce the reality and it structurally gives negative answers on the effectiveness of the therapy. These reasons, combined with the possible low-level oscillations of model (1) makes again stochastic effects worth investigating. Along the line of [39], this shall permit to have an estimation of the average value of the random therapy-induced eradication time *t_er _*better than the one obtainable in the deterministic framework, that is better than min{*t *| *T *(*t*) *<*1} which would be actually very rough. As in [39], switching from the deterministic to the hybrid version of the model is furthermore justified by the fact that, at the best of our knowledge, there is no proof that the average of the state variables of a nonlinear birth and death stochastic processes follows the dynamics of the corresponding ODE system, when variables assume low values. In fact, the ODE system would be a good approximation of the purely stochastic model if the state variables were "sufficiently large". In any case, by using the above deterministic approximation of *t_er _*its standard deviation could never be computed.

We now present an SSA-based algorithm to simulate the hybrid version of the above model. This new algorithm extends the algorithm which simulates model (2) in a natural way. The reactions in Table 1 are left unchanged provided that the propensity function for *R*_7 _is modified as *a*_7_(*t*) = *V σ_E _*(*t*) to reflect the time-dependency of *σ_E _*(*t*). As a consequence in this case we have two time-dependent propensity functions *a*_4 _(*t*) and *a*_7 _(*t*), hence we define $S=R\backslash \left\{R_{4},R_{7}\right\}$. Let us model the event-induced changes in the values of *T *and *E *by the vectors

$$\left(\begin{array}{c} \nu_{T}\\ \nu_{E} \end{array}\right)=\left(\begin{array}{ccccccc} 1 & -1 & -1 & 0 & 0 & 0 & 0\\ 0 & 0 & 0 & 1 & -1 & 1 & 1 \end{array}\right).$$

Here the *i*-th components of *ν_T _*and *ν_E_*, i.e. *ν*_*T*,*i *_and *ν*_*E*,*i*_, describe how the reaction *R_i _*affects the population *T *and *E*, respectively. With the last stochastic event fired at time *t_n _*we define

$$A_{4}\left(\tau \right)=\int_{t_{n}}^{t_{n}+\tau }a_{4}\left(t\right)dt$$

and

$$A_{7}\left(\tau \right)=\int_{t_{n}}^{t_{n}+\tau }a_{7}\left(t\right)dt=V\int_{t_{n}}^{t_{n}+\tau }\sigma_{E}\left(t\right)dt.$$

The exact simulation algorithm for the hybrid system with generic immunotherapies is Algorithm 1 and is defined in Table 2. With the current state **x **and the propensity functions in S evaluated, i.e. $a_{0}^{S}=\sum_{j\in S}a_{j}\left(\text{x}\right)$ the putative time for the next stochastic event follows

(5) $$A_{4}\left(\tau \right)+a_{0}^{S}\tau +A_{7}\left(\tau \right)=\chi$$

where *χ *is a random number with distribution *Exp*(1). Notice that equation (5) is a natural extension of equation (3) and, as such, it does not admit a general analytical solution. Consequently numerical methods to find its solution are again required. In the following, when looking for a solution of equation *f*(*x*) = 0 in [*a*, *b*] with *f*(*a*)*f*(*b*) *<*0 and *f *continuous we always adopt the *bisection method *with double stopping criteria |*b *- *a*| *<*10^-8 ^∧ |*f*(*x*)| *<*10^-6^. Once the jump is determined, given $a_{0}=a_{0}^{S}+a_{4}\left(t+\tau \right)+a_{7}\left(t+\tau \right)$, the next event to fire find is *R_j _*if

(6) $$\overset{j-1}{\underset{i=1}{\sum }}a_{i}\left(t+\tau \right)<r\cdot a_{0}\leq \overset{j}{\underset{i=1}{\sum }}a_{i}\left(t+\tau \right)$$

where *r *is a random number *U*[0, 1] and *a_i_*(*t *+ *τ *) = *a_i_*(**x**) if i∈S. It is important to notice that equations (5) and (6) would reduce to the standard equations used in the SSA if the system was entirely stochastic.

Some considerations about the ODEs constituting the model (4) are worth discussing. If the last stochastic event happened at time *t_n _*and the next will happen at time *t*_*n*+1 _the equation for *I*' reads as

(7) $$I^{\prime }=\frac{p_{I}}{V}\frac{T\left(t_{n}\right)E\left(t_{n}\right)}{g_{I}V+T\left(t_{n}\right)}+\sigma_{I}\left(t\right)-\mu_{I}I$$

which, since in such intervals the other two state variables are constant, is a linear ODE with constant input and constant coefficients. Thus, given *I*(*t_n_*) = *I_n _*its analytical solution in (*t*_*n*_, *t*_*n*+1_) is

(8) $$\begin{array}{llll} I\left(t\right)=B_{n} & +\left(I_{n}-B_{n}\right)e^{-\mu_{I}\left(t-t_{n}\right)}\; & & \;\\ & +\;e^{-\mu_{I}t}\int_{t_{n}}^{t}\sigma_{I}\left(z\right)e^{\mu_{I^{Z}}}dz\; & & \;\\ & \; & \end{array}$$

where

(9) $$B_{n}=\frac{1}{\mu_{I}}\left(\frac{p_{I}}{V}\frac{T\left(t_{n}\right)E\left(t_{n}\right)}{g_{I}V+T\left(t_{n}\right)}\right).$$

Notice that equation (8) is necessary to evaluate, at each step of Algorithm 1, the value of *a*_4_(*t*). In the next sections we will consider specific types of immunotherapies and, according to their mathematical modeling, we may discuss on equation (8).

**Table 2:** The Hybrid Simulation Algorithm (Algorithm 1)

**Require: **(*T*_0_, *E*_0_, *I*_0_), *t*_0_, *t_stop_*.
1: set the initial state to (*T*_0_, *E*_0_, *I*(*t*_0_)) and the initial time *t *to *t*_0_;
2: **while ***t < t_stop _***do**
3: let **x **be the current state, for j∈S evaluate *a_j_*(**x**), define $a_{0}^{S}=\sum_{j\in S}a_{j}\left(x\right)$;
4: let *χ *be a random number with distribution *Exp*(1), solve the transcendental equation
$A_{4}\left(\tau \right)+a_{0}^{S}\tau +A_{7}\left(\tau \right)=\chi$
and then define $a_{0}=a_{0}^{S}+a_{4}\left(t+\tau \right)+a_{7}\left(t+\tau \right)$;
5: let *r *be a random number *U*[0, 1], for the next event to fire find *j *by solving
$\overset{j-1}{\underset{i=1}{\sum }}a_{i}\left(t+\tau \right)<r\cdot a_{0}\leq \overset{j}{\underset{i=1}{\sum }}a_{i}\left(t+\tau \right)$
where if i∈S then *a_i_*(*t *+ *τ *) = *a_i_*(**x**);
6: update (*T*, *E*, *I*(*t*)) to (*T *+ *ν*_*T*,*j*_, *E *+ *ν*_*E*,*j*_, *I*(*t *+ *τ *)) and change clock to *t *+ *τ *;
7: **end while**

Input: initial state (*T*_0_, *E*_0_, *I*_0_), start time *t*_0_, stop time *t_stop_*.

### Values of the parameters

We discuss now the values of the parameters used to simulate the model. In Table 3 we recall the parameters used in [39] to perform the simulations. Those values and ranges are originally given in [14,48]. Note that those values pertain to mice and they were taken from [20,49], where accurate fitting of real data concerning laboratory animals was performed. As in [39] volume *V *is estimated to be 3.2 *ml*. This follows by the body weight of a femal chimeric mouse ranging from 20 to 40 *grams*, and by considering also that their blood volume ranges from 5.8 to 8 *ml *per 100 *grams*. It follows that *V *reasonable ranges from 1.16 up to 3.2 *ml*. We remark that we will give the parameters for the functions *σ_I_*(*t*) and *σ_E_*(*t*) in the next sections when the therapies will be discussed.

**Table 3:** Values of the parameters

**Par**.	Value	Unit	Description
*r*	0.18	*days*^-1^	baseline growth rate of the tumor
*b*	10^-9^	*ml*^-1^	carrying capacity of the tumor
*a*	1	*ml*/*days*	baseline strength of the killing rate by immune effectors
*c*	10^-4^	*days*^-1^	tumor antigenicity
*V*	3.2	*Ml*	blood and bone marrow volumes for leukemia
*g_T_*	10^5^	*ml*^-1^	50% reduction factor of the killing rate by immune effectors
*g_E_*	2 · 10^7^	*pg*/*l*	50% reduction factor of IL-stimulated growth rate of effectors
*g_I_*	10^3^	*ml*^-1^	50% reduction factor of production rate of interleukins
*p_E_*	0.1245	*days*^-1^	baseline strength of the IL-stimulated growth rate of effectors
*p_I_*	5	*pg*/*days*	baseline strength of production rate of interleukins
*μ_E_*	0.03	*days*^-1^	inverse of average lifespan of effectors
*μ_I_*	10	*days*^-1^	loss/degradation rate of *IL*_2_

Parameters of model (4), as given in [39].

### Interleukin-based immunotherapies

In the next paragraphs we discuss how to specialize model (4) with either a piece-wise constant or an impulsive interleukin-based immunotherapy. In both cases, we consider a therapy starting at time *t_s_*, ending at time *t_e _*and consisting of the injection of molecules of IL-2. As already said a really uninterrupted continuous infusion therapy in [*t_s_*, *t_e_*], such as those in [14,39], is not realistic. Here we consider a continuous infusion therapy delivered at pre-set times

$$\Theta =\left\{\theta_{i}|i=0,\ldots ,k\right\}$$

where *θ_i _*∈ [*t_s_*, *t_e_*] for *i *= 0, . . . , *k*. In the following, with a slight abuse of terminology, we refer to each *θ_i _*∈ Θ as a *therapy session*.

#### Piece-wise constant therapy

In the case of a continuous infusion each therapy session has a duration of *A *time-units and a constant influx rate *d_i _*in the *i*-th infusion. We model the therapy as

(10) $$\sigma_{I}\left(t\right)=\left\{\begin{array}{cc} 0 & \text{if}\;t_{s}<t<t_{e}\\ \sum_{i=0}^{k}d_{i}g\left(t-\theta_{i},\;A\right) & \text{otherwise} \end{array}\right)$$

where *A <*min{*θ*_*i*+1 _- *θ*_*i *_| *i *= 0, . . . , *k *- 1} (i.e. no overlap between two therapy sessions), and *g*(*t*, *A*) is the "window function" of amplitude *A*, i.e.

g(t,A)=H(t)-H(t-A)

where *H*(·) the well-known Heavyside function. Although this scenario can be simulated by Algorithm 1, some considerations about the equations that need to be solved are worth discussing. In particular, when solving for *t *>*t_n _*equation (8) by using equation (10) the key point is to evaluate the integration part of Equation (8), i.e.

$$R\left(t_{n},t\right)=\int_{t_{n}}^{t}\sigma_{I}\left(x\right)e^{\mu_{I}x}dx=\int_{t_{n}}^{t_{n}+\varphi }\eta \left(x\right)dx$$

where *t *= *t_n _*+ *φ *and *η *(*x*) = *σ_I_*(*x*)*e^μIx^*. Since Θ = {*θ*_0_, *θ*_1_, . . . , *θ_k_*}, we start by considering the set of therapy sessions which start and complete in the time window (*t_n_*, *t*_*n *_+ *φ*), such a set is

$$\theta \left(t_{n},\varphi \right)=\left\{\theta_{j}\in \Theta |t_{n}<\theta_{j}\wedge \theta_{j}+A<t_{n}+\varphi \right\}.$$

Now, since Θ contains ordered time-points then also *θ*(*t*_*n*_, *φ*) does. We want to get the index of the minimum and the maximum *θ_j_*'s from *θ*(*t*_*n*_, *φ*); we define

$$\begin{array}{c} \theta_{min}=\min\left(\theta \left(t_{n},\varphi \right)\right)\\ \theta_{max}=\max\left(\theta \left(t_{n},\varphi \right)\right) \end{array}$$

so that the indexes *min *and *max *denote such values. To solve *R*(*t_n_*, *t*_*n *_+ *φ*) we split the integral in three time intervals [*t_n_*, *θ_min_*), [*θ_min_*, *θ_max _*+ *A*] and (*θ_max _*+ *A*, *t_n _*+ *φ*] so that

$$\begin{array}{llll} R(t_{n},t_{n} & +\varphi )=\int_{t_{n}}^{\theta_{min}}\eta \left(x\right)dx\; & & \;\\ & +\int_{\theta_{min}}^{\theta_{max}+A}\eta \left(x\right)dx+\int_{\theta_{max}+A}^{t_{n}+\varphi }\eta \left(x\right)dx.\; & & \;\\ & \; & \end{array}$$

The integral in [*θ_min_*, *θ_max _*+ *A*] is the summation of |*θ*(*t*_*n*_, *φ*)| non-zero basic integrals over the sub-intervals [*θ_j_*, *θ_j _*+ *A*] for any *θ_j _*∈ *θ*(*t*_*n*_, *φ*), that is

$$\int_{\theta_{min}}^{\theta_{max}+A}\eta \left(x\right)dx=\underset{\theta_{j}\in \theta \left(t_{n},\varphi \right)}{\sum }\left(\int_{\theta_{j}}^{\theta_{j}+A}g\left(x\right)dx\right).$$

Notice that for any *θ_j _*∈ *θ*(*t*_*n*_, *φ*) it holds that *σ_I_*(*x*) = 0 for any *x *∈ (*θ_j _*+ *A*, *θ*_*j*+1_) (i.e. when the therapy is not delivered) yielding $\int_{\theta_{j}+A}^{\theta_{j+1}}\eta \left(x\right)dx=0$. Furthermore, since

$$\begin{array}{llll} \int_{\theta_{j}}^{\theta_{j}+A}\sigma_{I}\left(x\right)e^{\mu_{I}x}dx & =\frac{d_{j}}{\mu_{I}}{\left[e^{\mu_{I}x}\right]}_{\theta_{j}}^{\theta_{j}+A}\; & & \;\\ & =\frac{d_{j}e^{\mu_{I}\theta_{j}}\left(e^{\mu_{I}A}-1\right)}{\mu_{I}}\; & & \;\\ & \; & \end{array}$$

then the overall integral evaluates as

$$\int_{\theta_{min}}^{\theta_{max}+A}\eta \left(x\right)dx=\frac{e^{\mu_{I}A}-1}{\mu_{I}}\left(\underset{\theta_{j}\in \theta \left(t_{n},\varphi \right)}{\sum }d_{j}e^{\mu_{I}\theta_{j}}\right).$$

Notice that this quantity can be easily computed in a iterative fashion. The cases of the rightmost and leftmost integration intervals are similarly accounted. Let us consider the leftmost interval [*t_n_*, *θ_min_*), we consider whether *t_n _*is included in in the (*min *- 1)-th therapy-session. We have this definition

$$\int_{t_{n}}^{\theta_{min}}\eta \left(x\right)dx=\frac{d_{min-1}}{\mu_{I}}\left(e^{\mu_{I}\left(\theta_{min-1}+A\right)}-e^{\mu_{I}t_{n}}\right)$$

if *θ*_*min*-1 _*< t_n _*≤ *θ*_*min*-1 _+ *A *and 0 otherwise. This holds since in the uppermost case $\int_{t_{n}}^{\theta_{min}}\eta \left(x\right)dx=\int_{t_{n}}^{\theta_{min-1}+A}\eta \left(x\right)dx$. Similarly, in the interval (*θ_max_*, *t_n _*+ *φ*] by cases on the relation between *t_n _*+ *φ *and *θ*_*max*+1_

$$\int_{\theta_{max}+A}^{t_{n}+\varphi }\eta \left(x\right)dx=\frac{d_{max+1}}{\mu_{I}}\left(e^{-\mu_{I}t}-e^{\mu_{I}\theta_{max+1}}\right)$$

if *θ*_*max*+1 _≤ *t_n _*+ *φ < θ*_*max*+1 _+ *A *and 0 otherwise. This holds since in the uppermost case $\int_{\theta_{max}+A}^{t_{n}+\varphi }\eta \left(x\right)dx=\int_{\theta_{max+1}}^{t_{n}+\varphi }\eta \left(x\right)dx$. We remark that all these combinations of cases are necessary because of all the possible combinations of the parameters *t *and *t_n _*with the set Θ.

#### Impulsive therapy

In many cases the infusions are very short implying that the in flux rate reaches very large values, so that one may approximate *σ_I_*(*t*) as a train of pulses, i.e.

(11) $$\sigma_{I}\left(t\right)=\left\{\begin{array}{cc} 0 & \text{if}\;t_{s}<t<t_{e}\\ \sum_{i=0}^{N}u_{i}\delta \left(t-\theta_{i}\right), & \text{otherwise}. \end{array}\right)$$

Here, *u_i _*is the *i*-th injected dose of molecules of IL-2 and *δ*(*t *- *θ_i_*) is the Dirac's delta function centered at *t *= *θ_i_*. By simple algebraic manipulations it is possible to see that here function *R*(*t_n_*, *t*) is given by

$$R\left(t_{n},t\right)=\overset{i_{e}}{\underset{i=i_{s}}{\sum }}u_{i}e^{\mu_{I}\theta_{i}}H\left(t-\theta_{i}\right)$$

where $\left\{\theta_{i_{s}},\ldots ,\theta_{i_{e}}\right\}=\Theta \cap \left(t_{n},t\right)$. Finally, we stress that an alternative to the use of the Dirac's generalized functions is to represent the drug deliveries as impulsive kicks given to *I*(*t*), thus becoming an impulsive differential equation [50,51]. Indeed, we have that at time *θ_i _*∈ Θ the value of *I*(*θ_i_*) increases by *u_i _*units thus yielding

(12) $$I\left(\theta_{i}^{+}\right)=I\left(\theta_{i}^{-}\right)+u_{i}$$

for *i *= 0, . . . , *k*.

### Adoptive cellular immunotherapies

In the next paragraphs we discuss how to specialize model (4) with either a piece-wise constant or an impulsive ACI. As in the previous section we consider a set of *k *therapy sessions Θ = {*θ_i _*| *i *= 0, . . . , *k*}.

#### Piece-wise constant therapy

As before we assume

(13) $$\sigma_{E}\left(t\right)=\left\{\begin{array}{cc} 0 & \text{if}\;t_{s}<t<t_{e}\\ \sum_{i=0}^{k}d_{i}g\left(t-\theta_{i},A\right) & \text{otherwise}. \end{array}\right)$$

This scenario can be simulated by Algorithm 1 where, in this case, the function *A*_7_(*τ*) is given by

$$\begin{array}{llll} A_{7}\left(\tau \right) & =V\Psi \left(t\right)+V\overset{i_{e}}{\underset{i=i_{s}}{\sum }}d_{i}\left(t-\theta_{i}\right)H\left(t-\theta_{i}\right)\; & & \;\\ & -\;V\overset{i_{e}}{\underset{i=i_{s}}{\sum }}d_{i}\left(t-\theta_{i}-A\right)H\left(t-\theta_{i}-A\right)\; & & \;\\ & \; & \end{array}$$

where $\left\{\theta_{i_{s}},\ldots ,\theta_{i_{e}}\right\}=\Theta \cap \left(t_{n},\;t\right)$ and

$$\begin{array}{llll} \Psi \left(t\right) & =H\left(\theta_{i_{s}-1}+A-t_{n}\right)d_{i_{s}-1}\left[\left(t-t_{n}\right)H\left(t-\theta_{i_{s}-1}\right)\right)\; & & \;\\ & -\left(\left(t-\theta_{i_{s}-1}-A\right)H\left(t-\theta_{i_{s}-1}-A\right)\right].\; & & \;\\ & \; & \end{array}$$

#### Impulsive therapy

We consider the case of an impulsive ACI where at each *θ_i _*∈ Θ there is the rapid infusion of *w_i _*cultured effectors. Thus, we could proceed similarly to the case of impulsive IL-based therapy. However, here the introduction of generalized functions does not lead to simplifications, and as a consequence we shall model the therapy as

(14) $$E\left(\theta_{i}^{+}\right)=E\left(\theta_{i}^{-}\right)+w_{i}.$$

Thus to the "natural" stochastic events external deterministic events are superimposed. As a consequence, at such times (*i*) the differential equation ruling the dynamics of *I*(*t*) must be updated and (*ii*) all the propensity functions involving *E *change value. This means that the integral terms of equation (5) in Algorithm 1 should be modified to consider such changes. However, results in [52] allow us for a modification of the algorithm that we discuss now. One of the fundamental properties of the SSA is that, with the system at time *t*, for any possible value of *τ *the propensity functions are constant in the time interval [*t*, *t *+ *τ*). The same holds in some configurations of the hybrid model studied in [39]. However, this does not hold in the case of impulsive ACI since, if *t_n _*is the time of the *n*-th stochastic event and *θ_j _*>*t_n _*is the closest injection after *t_n_*, the propensity functions *a*_3 _and *a*_5 _change after *θ_j_*, invalidating the property. Of course, the time-dependent propensity functions *a*_4 _and *a*_7 _are, by definition, potentially non-constant. We argue that there is a similarity between scheduling-based SSAs for systems with delays and this hybrid system (i.e. consider this ACI as a set of events scheduled at the preset times in Θ). Although this differs from such algorithms where the scheduling times are stochastically chosen during the simulation, this allows to modify Algorithm 1 into Algorithm 2 presented in Table 4, which is indeed inspired by the algorithms discussed in [53-57]. As for such algorithms, the correctness of Algorithm 2 relies on the existence of a general schema of SSA-based algorithm with piece-wise constant time-dependent propensity functions [52] where is stated tha, and this algorithm respects that schema.

**Table 4:** The Hybrid Simulation Algorithm (Algorithm 2)

**Require: **(*T*_0_, *E*_0_, *I*_0_), *t*_0_, *t_stop_*.
1: initialize the simulation as for Algorithm 1;
2: **while ***t < t_stop _***do**
3: pick a value for *τ *as in Algorithm 1;
4: get *θ_next _*= min{*θ_i _*∈ Θ | *θ_i _*>*t*}
5: **if ***τ < θ_next _***then**
6: fire a reaction as in Algorithm 1;
7: **else**
8: update (*T*, *E*, *I*(*t*)) to (*T*, *E *+ *w_next_*, *I*(*t *+ *θ_next_*)) and change clock to *t *+ *θ_next_*;
9: **end if**
10: **end while**

Input: initial state (*T*_0_, *E*_0_, *I*_0_), start time *t*_0_, stop time *t_stop_*.

### Combining IL-2 therapies and ACI

Combining therapies requires combining the results of the previous sections. To shorten the presentation we briefly discuss how to perform the simulations of the hybrid model with combined immunotherapies.

Whenever an impulsive ACI is considered, independently of the IL-2 therapy, the model can be simulated by Algorithm 2. In the other cases the model can be simulated by Algorithm 1.

## Results

In the next sections we perform both asymptotic and transitory analysis of the solutions of deterministic system (4). We show the existence of deterministic conditions that guarantee the eradication of the tumor, and that will be used to tune the parameters of our hybrid model when performing its simulations, which are discussed in the forthcoming section.

### Deterministic asymptotic analysis

With the aid of elementary dynamical systems theory [41] and by using some mathematical properties summarized in the additional file 1, here we briefly investigate how the therapies may influence the asymptotic behavior of the solutions of the deterministic system (4). We shall focus on the mathematically idealized case of infinite length of the therapy and on the deterministic conditions guaranteeing the eradication of the tumor.

#### IL-2 immunotherapy

We start from the case of the delivery of a IL-based mono-therapy (i.e. *σ_E_*(*t*) = 0). By setting *T *= *E *= 0 it follows *I*(*t*) = *J*_∞_(*t*), where *J*_∞_(*t*) is the asymptotic solution of

(15) $$J^{\prime }=-\mu_{I}J+\sigma_{I}\left(t\right).$$

Thus, we found a tumor-free state

$$TF_{I}=\left(0,0,J_{\infty }\left(t\right)\right)$$

which is, however, unstable. In fact, this follows by setting

$$\left(T,E,I\right)=\left(0,0,J_{\infty }\left(t\right)\right)+\left(T_{l},E_{l},I_{l}\right),$$

and linearizing since the equation for tumor cells reads as $T_{l}^{\prime }=rT_{l}$. Even though this means that the tumor-free state (0, 0, *J*_∞_(*t*)) is unstable, this does not mean that under an IL-based mono-therapy a tumor cannot be eradicated. In fact, as we show, there exist conditions implying that (*T*, *E*, *I*) → (0, +∞, +∞), namely the tumour is eradicated. This can be verified by using basic differential inequalities recalled in the additional file 1. Indeed, from the differential inequality

$$I^{\prime }\geq -\mu_{I}I+\sigma_{I}\left(t\right)$$

it follows that *I*(*t*) >*J*(*t*) and, for large times, *I*(*t*) ≥ *J*_∞_(*t*). In turn, the inequality

$$\frac{p_{E}I\left(t\right)}{g_{E}+I\left(t\right)}>\frac{p_{E}J_{\infty }\left(t\right)}{g_{E}+J_{\infty }\left(t\right)}$$

yields the inequality

(16) $$E^{\prime }\geq \left(\frac{p_{E}J_{\infty }\left(t\right)}{g_{E}+J_{\infty }\left(t\right)}-\mu_{E}\right)E.$$

Finally, from the properties of periodical linear differential equations recalled the additional file 1 we can observe that

(17) $$p_{E}\left\langle \frac{J_{\infty }\left(t\right)}{g_{E}+J_{\infty }\left(t\right)}\right\rangle \geq \mu_{E}$$

implies that (*T*(*t*), *E*(*t*)) → (0, +∞). In other words, the deterministic model predicts that eradication is possible only at the price of killing the patient for the excess of stimulation of the immune system, that is *E*(*t*) → +∞. We remark that the above inference does not consider finite length therapies. In fact, if at the end of the therapy the tumor has been eradicated, the number of effectors and the interleukin density will both decay. Inequality (17) is the condition which we obtain by the deterministic model. We now refine such a condition to account for the three therapies that we mentioned so far: (*i*) constant, (*ii*) piece-wise constant and (*iii*) impulsive. It holds that:

(*i*) when a constant therapy where *σ_I_*(*t*) = *σ_I _*is considered *J*_∞ _= *σ_I _*/*μ_I _*and condition (17) is

$$\frac{p_{E}\sigma_{I}}{g_{E}\mu_{I}+\sigma_{I}}\geq \mu_{E}.$$

(*ii*) if we consider the piece-wise constant *σ_I_*(*t*) of equation (10) where the therapy sessions are periodically scheduled with period *P*, duration *A *and the rate of each session is the same, that is ∀*θ_i _*∈ Θ. *d_i _*= *d*, we have that

(18) $$J_{\infty }\left(t\right)=\left\{\begin{array}{cc} \frac{d}{\mu_{I}}-\beta_{1}e^{-\mu_{I}t} & \text{if}0\leq t<A\\ \beta_{2}e^{-\mu_{I}\left(t-A\right)} & \text{if}\;0\leq t<P \end{array}\right)$$

where

$$\beta_{1}=\frac{d}{\mu_{I}}\frac{e^{\mu_{I}P}-e^{\mu_{I}A}}{e^{\mu_{I}P}-1}\;\beta_{2}=\frac{d}{\mu_{I}}-\beta_{1}e^{-\mu_{I}A}.$$

In this case condition (17) reads as

$$\frac{p_{E}}{P}\left(1-\omega_{1}-\omega_{2}\right)\geq \mu_{E}$$

where

$$\begin{array}{c} \omega_{1}=\frac{g_{E}}{\mu_{I}\left(g_{E}+\alpha_{1}\right)}\log\left(\frac{e^{\mu_{I}A}-\gamma_{1}}{1-\gamma_{1}}\right)\\ \omega_{1}=\frac{1}{\mu_{I}}\log\left(\frac{e^{\mu_{I}\left(P-A\right)}+\gamma_{2}}{1+\gamma_{2}}\right)\\ \gamma_{1}=\frac{\beta_{1}}{g_{E}+\alpha_{1}}\;\;\;\gamma_{2}=\frac{\beta_{2}}{g_{E}} \end{array}$$

and *γ*_1 _*<*1.

(*iii*) if we consider the impulsive *σ_I_*(*t*) of equation (11) where all the therapy sessions are periodically scheduled with period *P *and the rate of each session is ∀*θ_i _*∈ Θ.*u_i _*= *U *we have

$$J_{\infty }\left(t\right)=\frac{U}{1-e^{-\mu_{I}P}}e^{-\mu_{I}\left(t\;\text{mod}\;P\right)}$$

where *t *mod *P *is the standard modulo operation. Then the eradication condition (17) is

(19) $$\frac{p_{E}}{P\mu_{I}}\log\left(\frac{ue^{\mu_{I}P}+g_{E}\left(e^{\mu_{I}P}-1\right)}{u+g_{E}\left(e^{\mu_{I}P}-1\right)}\right)>\mu_{E}.$$

#### ACI

When only ACI is delivered (i.e. *σ_I_*(*t*) = 0) there is the following tumor-free asymptotic solution

$$\left(0,\varepsilon_{E}\left(t\right),0\right)$$

where *ε_E_*(*t*) is the asymptotic solution of

(20) $$\varepsilon^{\prime }\left(t\right)=-\mu_{E}\varepsilon +\sigma_{E}\left(t\right)$$

which is the equation for *E *when *T *= *I *= 0. In the case of periodically delivered therapy with period *P*, the linearized equation for tumor cells is

$$T_{l}^{\prime }=\left(r-\frac{a}{g_{T}V}\varepsilon_{E}\left(t\right)\right)\tau_{\iota }$$

and hence the local eradication condition (17) is

(21) $$\frac{a}{g_{T}V}\left\langle \varepsilon E\left(t\right)\right\rangle \geq r.$$

However, by averaging both the sides of (20) yields

$$\varepsilon_{E}\left(P\right)-\varepsilon_{E}\left(0\right)=0=-\mu_{E}\left\langle \varepsilon_{E}\left(t\right)\right\rangle +\sigma_{E}\left(t\right)$$

which implies that

$$\left\langle \varepsilon_{E}\left(t\right)\right\rangle =\frac{\left\langle \sigma_{E}\left(t\right)\right\rangle }{\mu_{E}}.$$

Finally, in this case the local stability condition corresponding to equation (17) becomes

(22) $$\frac{a}{g_{T}V}\frac{\left\langle \sigma_{E}\left(t\right)\right\rangle }{\mu_{E}}\geq r.$$

As for the case of IL-2 mono-therapy we now focus on the therapies considered so far, we have that:

(*i*) if we consider constant *σ_E_*(*t*) then condition (22) is *σ_E _*>*rg_T _V/a*.

(*ii*) if we consider a piece-wise constant *σ_E_*(*t*) with infinite length *t_e _*= +∞, period *P *where *θ_i _*= *iP*, duration *A *and injection rate *b_i _*= *b *for any *θ_i _*∈ Θ, then similarly to the case of the IL-based mono-therapy one can show that

$$\varepsilon_{\infty }\left(t\right)=\frac{b}{\mu_{E}}-\frac{b}{\mu_{E}}\xi e^{-\mu_{E}t}$$

where

$$\xi =\frac{e^{\mu_{E}P}-e^{\mu_{E}A}}{e^{\mu_{E}P}-1}$$

if 0 ≤ *t < A *and

$$\varepsilon_{\infty }\left(t\right)=\left(\frac{b}{\mu_{E}}-\frac{b}{\mu_{E}}\xi e^{-\mu_{E}A}\right)e^{-\mu_{E}\left(t-A\right)}$$

if 0 ≤ *t < P *and the eradication condition can be consequently computed.

(*iii*) if we consider the impulsive therapy *σ_E_*(*t*) where the common injection rate is *w_i _*= *w *for *i *= 1, . . . , +∞, by using the results reported in the additional file 1 we have that

$$\varepsilon_{E}\left(t\right)=\frac{w}{1-e^{-\mu_{E}P}}e^{-\mu_{E}P}$$

and hence the eradication condition (22) is

(23) $$\frac{a}{g_{T}V}\frac{w}{P\mu_{E}}\geq r.$$

#### Combined therapies

Finally, we shortly consider when both the therapies are delivered. In this case there is the following tumor-free asymptotic solution

$$\left(0,\varepsilon_{\infty }\left(t\right),J_{\infty }\left(t\right)\right)$$

where *ε*_∞_(*t*) is the asymptotic solution of

(24) $$\varepsilon^{\prime }\left(t\right)=\left(\frac{p_{E}J_{\infty }\left(t\right)}{g_{E}+J_{\infty }\left(t\right)}-\mu_{E}\right)\varepsilon +\sigma_{E}\left(t\right).$$

In the case of synchronous delivery with common period *P *the local eradication condition becomes

(25) $$\frac{a}{g_{T}V}\left\langle \varepsilon_{\infty }\left(t\right)\right\rangle \geq r.$$

As for the mono-thrapeutic case in some of the scenarios that we mentioned it is possible to infer analytical local eradication conditions. In the additional file 1 we show the derivation of the eradication condition for combined impulsive therapies with synchronous delivery.

#### Global stability of the eradication

We conclude by investing the global stability of the eradication. Since for sufficiently large *t *it is *I*(*t*) ≥ *J*_∞_(*t*) then from the differential inequality

$$E^{\prime }\left(t\right)>\left(\frac{p_{E}J_{\infty }\left(t\right)}{g_{E}+J_{\infty }\left(t\right)}-\mu_{E}\right)E+\sigma_{E}\left(t\right)$$

follows that, for large times, *E*(*t*) ≥ *ε*_∞_(*t*). Then by

$$T^{\prime }<rT\left(1-\frac{b}{V}T\right)-\frac{aT}{g_{T}V+T}\varepsilon_{\infty }\left(t\right)$$

it follows that *T*(*t*) *< X*(*t*), where

$$X^{\prime }=rX\left(1-\frac{b}{V}X\right)-\frac{aX}{g_{T}V+x}\varepsilon_{\infty }\left(t\right).$$

Finally, it follows that if ∀*x*(0) > 0. *X*(*t*) → 0 then also *T*(*t*) → 0, and the tumor eradication is globally asymptotically stable [41].

### Deterministic transitory analysis

Results of the previous section refer to the highly idealized case of a infinite horizon therapy. However, real therapies have a finite duration and, more important, the host organism has a finite lifespan. Thus, in this as well as in other applications of computational biology and medicine it is natural to wonder whether such results can be used at all [8,28]. This is critically related to the velocity at which the solutions of the equations studied in the previous section tend to their asymptotic solutions.

As far as the IL-2 mono-therapy is concerned, the velocity of growth of *E*(*t*) - which in turn determines the velocity of reduction of *T*(*t*) - is ruled by the difference *p_E_*〈*J*_∞_(*t*)/(*g_E _*+ *J*_∞_(*t*))〉 - *μ_E_*. Moreover, independently of the initial conditions the function *J*(*t*) converges to *J*_∞_(*t*) in some multiple of average degradation time of the interleukin, i.e. 1/*μ_I_*, which is small. Thus, this means that very soon the asymptotic solution is reached. Observe now that since *J*_∞_(*t*)/(*g_E _*+ *J*_∞_(*t*)) *<*1 it follows that if *p_E _< μ_E _*then the constraint (17) is never fulfilled. We stress that this is the case for the values listed in Table 3. In practice, since in general 〈*J*_∞_(*t*)/(*g_E _*+ *J*_∞_(*t*))〉 *<<*1 and since *μ_E _*is small, it follows that *p_E _*must be far larger than *μ_E_*. This requires that, for some of the settings that we will simulate in the next sections, the value of *p_E _*could be different from the one given in Table 3. Biologically, this might substantially reduce the number of patients to whom the IL-2 mono-therapy might be effective.

Further discussions are worth. In the case of impulsive therapy unless IL-2 is injected every few hours *J*_∞_(*t*) → 0 rapidly, so that the eradication is unlikely unless large doses are delivered. This is also mirrored in the corresponding local eradication condition (19). Furthermore, in equation (16) it is also very important parameter *g_E_*, whose value used in our simulations is very large. Thus, if *μ_I_P *and *g_E _*are large we may roughly say that - unless huge doses are delivered or *p_E _*is particularly large - the coefficient of *E *in inequality (16) is almost always comparable to -*μ_E_*, so that a large rate of injection is required to fulfill eradication condition (19).

In the case of piece-wise continuous delivery of IL-2 *J*_∞_(*t*) takes few hours to get sufficiently closer its maximum plateau value, as given by equation (18). This suggests that for this kind of drug delivery the duration of each therapy session, i.e. *A*, should be a quite large fraction of the unity. This, of course, poses some practical problems since the patients should receive very long daily infusions. However, in some recent clinical trials on cyrcadian rythms-tuned delivery of chemotherapy some special 24-hours infusors have been experimented [58]. Roughly speaking, the above fact might be related to the "indirect effect" of IL-based therapies. In fact, they aim at triggering the expansion of the number of effector cells which, in turn, kill tumor cells.

Differently, as far as the ACI mono-therapy is concerned, the velocity of convergence of *ε_E_*(*t*) is some multiple of the average lifespan of the effector, i.e. 1/*μ_E_*. Such a value is generally quite big and, for instance, it is 33 days about in our simulations. This means that the convergence is very slow and that, unless the duration of the therapy is exceptionally long, the results of the asymptotic analysis cannot be used as a basis for the stochastic simulations. Similar considerations can be done for the combined therapy.

Finally, it is important to recall that the conditions that derived in the previous section are of local nature. In order to guarantee the eradication for generic non-small initial conditions (*T*(0), *E*(0), *I*(0)) the constraint specific to the simulated therapy has to be largely fulfilled.

### Stochastic simulations

We performed stochastic simulations of the model under various therapeutic settings, whose results are now reported. We have mostly considered a single-month daily therapy, i.e. Θ = {1, . . . , 30}. When different schedules are considered the parameters are explicitly reported. In all the figures representing simulation *days *and *number *of cells are given on the *x*-axis and the *y*-axis, respectively. To perform simulations a JAVA implementation of model (4) and Algorithms 1 and 2 has been developed.

#### IL-2 immunotherapy

**Figure 1:**
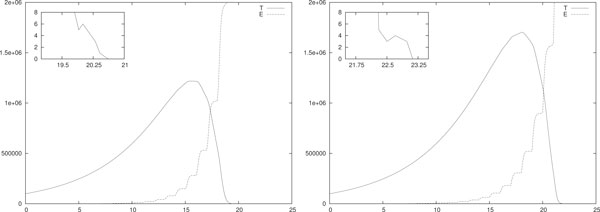
**Single run, IL-2 mono-therapy**. Single-run of piece-wise constant (left) and impulsive (right) IL-2 daily immunotherapy. In (left) *A *= 0.2 and *d_i _*= 4 · 10^7^/*A *for *i *= 1, . . . , 30. In (right) *u_i _*= 4 · 10^7 ^for *i *= 1, . . . , 30. In both cases *T*(0) = 10^5^, *E*(0) = *I*(0) = 0 and *p_E _*= 20 · 0.1245. All the other parameters are as in Table 3.

In Figure 1 a single stochastic run of the hybrid model with only IL-2 based immunotherapies is shown. We simulated both piece-wise constant (left) and impulsive (right) IL-2 daily immunotherapies with initial configuration *T*(0) = 10^5 ^and *E*(0) = *I*(0) = 0. In the former case each therapy session lasts *A *= 0.2 days, i.e. 4.8 hours, and *d_i _*= 4 · 10^7^/*A *for *i *= 1, . . . , 30. In the latter case at each therapy session the value *u_i _*for the injection is *u_i _*= 4·10^7 ^for *i *= 1, . . . , 30. Thus the total injected drug per day is the same in both cases. Moreover, according to the transitory analysis, in both cases we twentyfold increased the value of *p_E _*reported in Table 3, i.e. we used *p_E _*= 20 · 0.1245. As we already said, this requirement shall reduce the number of patients to whom this therapy might be effective. All the other parameters are as in Table 3.

Notice that (i.e. compare with the transitory analysis) the piece-wise constant immunotherapy seems more efficient than the impulsive one. Indeed (*i*) in the piece-wise case the eradication of the tumor is reached at *t_e _*≈ 20 days and the maximum tumor size is around 10^6 ^= 10 · *T*(0). Differently, (*ii*) in the impulsive case the tumor is eradicated a few days later, i.e. *t_e _*≈ 23 days, and the maximum tumor size is almost 20·*T*(0). At the eradication day the number of effector cells is around 4 · 10^6 ^in both cases, whereas the density of IL-2 is of the order of 10^7 ^in (left) and 10^4 ^in (right).

#### Adoptive Cellular Immunotherapy

**Figure 2:**
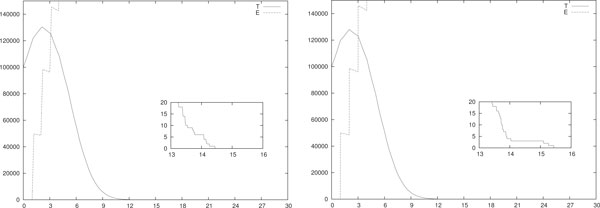
**Single run, daily ACI mono-therapy**. Single-run of piece-wise constant (left) and impulsive (right) daily ACI. In (left) *A *= 0.2 and *b_i _*= 5 · 10^4^/(*V A*) for *i *= 1, . . . , 30. In (right) *w_i _*= 5 · 10^4 ^for *i *= 1, . . . , 30. In both cases *T*(0) = 10^5 ^and *E*(0) = *I*(0) = 0. All the other parameters are as in Table 3.

In Figure 2 a single stochastic run of the hybrid model with only ACI is shown. As for IL-based therapy, we simulated both piece-wise constant (left) and impulsive (right) daily ACIs with initial configuration *T*(0) = 10^5 ^and *E*(0) = *I*(0) = 0. In the former case each therapy session lasts *A *= 0.2 days and *b_i _*= 25 · 10^4 ^for *i *= 1, . . . , 30. In the latter case at each therapy session 5·10^4 ^effector cells are injected, i.e. *w_i _*= 5 · 10^4 ^for *i *= 1, . . . , 30. Thus the number of injected effectors is equal in both cases. All the other parameters are as in Table 3.

Note that the figures show no remarkable difference in the tumor response. In particular, in both the simulations the eradication is obtained at around day 15. In both cases, at the eradication day the number of effector cells is around 6 · 10^5^, and the density of IL-2 is of the order of 10^2^.

**Figure 3:**
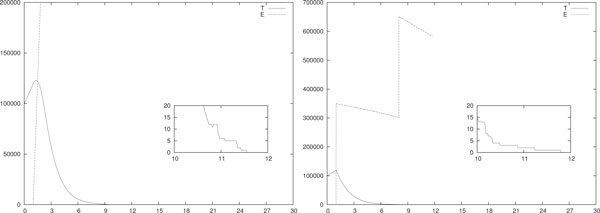
**Single run, weekly ACI mono-therapy**. Single-run of piece-wise constant (left) and impulsive (right) weekly ACI, i.e. in both panels *θ_i _*= 7*i *+ 1 for *i *= 0, . . . , 3. In (left) *A *= 1.4 days and *b_i _*= 35 · 10^4^/*V A i *= 0, . . . , 3. In (right) *w_i _*= 35 · 10^4 ^for *i *= 1, . . . , 3. All the other parameters are as in Table 3.

Finally, to discover the relation between the frequency of the therapy sessions and the dosage of each session, in Figure 3 a single stochastic run of the hybrid model with impulsive weekly ACI is shown. In those simulations we used an impulsive ACI with a weekly schedule (i.e. *θ_i _*= 7*i *+ 1 for *i *= 0, . . . , 3) with dosage *w_i _*= 35 · 10^4 ^for *i *= 1, . . . , 30. Note that this means that once a week a number of effectors is injected equivalent to the number of effectors given in an entire week of Figure 2 (right). The other parameters are as in Table 3.

Notice that it seems that the immune response is slightly better stimulated with this therapy setting than the one in Figure 2 (right). In fact, in this case the eradication day is around 12. The number of effector cells and the density of IL-2 are similar to those in Figure 2 (right).

#### Combined therapies

**Figure 4:**
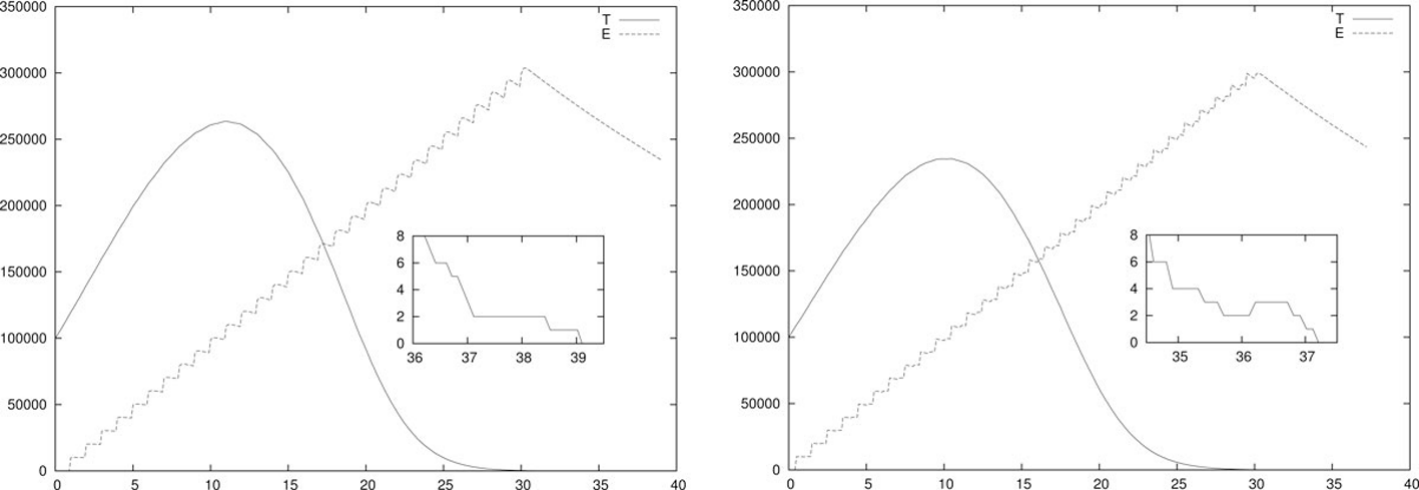
**Single run, combined immunotherapies**. Single-run of synchronous (left) and asynchronous (right) combined impulsive IL-2 and ACI daily immunotherapies. The asynchronous delivery is a shift of 0.5 days. In (left) *u_i _*= 10^6 ^for *i *= 1, . . . , 30 and in (right) *w_i _*= 10^4 ^for *i *= 1, . . . , 30. In both cases *T*(0) = 10^5^, *E*(0) = *I*(0) = 0 and *p_E _*= 20 · 0.1245. All the other parameters are as in Table 3.

In Figure 4 a single stochastic run of the hybrid model with combined impulsive IL-2 and ACI daily immunotherapies is shown. In (left) both the therapies are given at the same day (i.e. with the same Θ). In (right) the therapies are asynchronous with a shift of 0.5 days, i.e. $\Theta_{i}^{IL}=i+\text{0}.5$ and $\Theta_{i}^{E}=i$ for *i *= 1, . . . , 30. Again, the initial configuration is *T*(0) = 10^5^, *E*(0) = *I*(0) = 0. The parameters for the therapies dosage and duration are the same for both cases. As far as IL-2 therapy is concerned, at each therapy session the value *u_i _*for the injection is *u_i _*= 10^6 ^for *i *= 1, . . . , 30. Differently, in each ACI session 10^4 ^effector cells are injected, i.e. *w_i _*= 10^4 ^for *i *= 1, . . . , 30. Notice that both the values are smaller than those used in the corresponding mono-therapy scenarios of Figures 1 (right) and 2 (right). Moreover, as in the case of single IL-2 therapies the value of *p_E _*is set to *p_E _*= 20 · 0.1245. All the other parameters are as in Table 3.

As expected, this combined therapy eradicates even though the parameters are lower than those used in the scenarios where the single therapies are used (i.e. Figure 1 (right) and 2 (right)). In both cases the eradication is observed a few days after the end of the therapy. In the case of synchronous therapy *t_er _*≈ 39, in the asynchronous case *t_er _*≈ 37. In both cases at the day of eradication the number of effector cells is around 2 · 10^5 ^and the density of IL-2 is around 10. In both therapies the maximum size of the tumor is almost equal, i.e. it is around 2.5·10^5^. Finally, since in both cases the proliferation of effector cells is almost equal, it seems that no remarkable differences are observed with these therapy schedules.

#### Larger initial tumor and ACI

**Figure 5:**
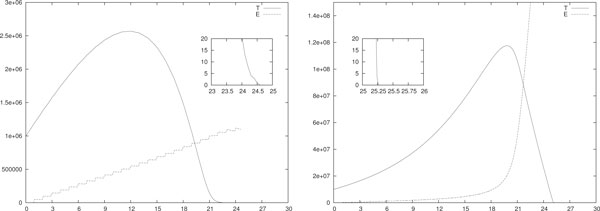
**Single run, ACI mono-therapy and larger tumor**. Single-run of impulsive daily ACI with *T*(0) = 10^6 ^(left) and *T*(0) = 10^7 ^(right). In both cases *E*(0) = *I*(0) = 0. In (left) *w_i _*= 5 · 10^4 ^for *i *= 1, . . . , 30. In (right) *w_i _*= 20 · 10^5 ^for *i *= 1, . . . , 30. All the other parameters are as in Table 3.

We analyzed the effect of varying the initial number of tumor cells in a scenario with impulsive ACI. Again, we analyzed this scenario because it was the one which permitted a computationally easier analysis. In Figure 5 a single stochastic run of the hybrid model with *T*(0) = 10^6 ^(left) and *T*(0) = 10^7 ^(right) is shown. In both cases *E*(0) = *I*(0) = 0. Notice that in this case *T*(0) is either 10 or 100 times larger than the value used in Figure 2. In left panel of Figure 5 at each therapy session 5 · 10^4 ^effector cells are injected, i.e. *w_i _*= 5 · 10^4 ^for *i *= 1, . . . , 30, and the eradication is reached at ≈ 24.5 *days*. On the contrary, in the case where *T*(0) = 10^7 ^and the same schedule is applied the eradication was not observed and the tumor size reached, quite rapidly, a size of the order of 10^9^. In order to obtain eradication also for *T*(0) = 10^7^, as shown in the right panel of Figure 5, we increased the number of effectors injected at each therapy session, i.e. *w_i _*= 20 · 10^4 ^for *i *= 1, . . . , 30 (i.e. each injection is 4 times bigger than the one shown in the left panel). In both cases all the other parameters are as in Table 3.

In both cases the eradication is observed close to the end of the therapy (i.e. day 25) with the number of effector cells being around 10^6 ^(left) and 10^8 ^(right), and the density of IL-2 of the order of 10^2 ^(left) and 10^7 ^(right). Notice that in (left) the line of the effectors is interrupted at the eradication time since the simulation is interrupted when *T *= 0. Moreover, Differently form the other figures here the scales on the *y*-axes are different since the maximum in (right) is 100 times bigger than the maximum in (left).

#### Probabilistic analysis of IL-2 therapy

**Figure 6:**
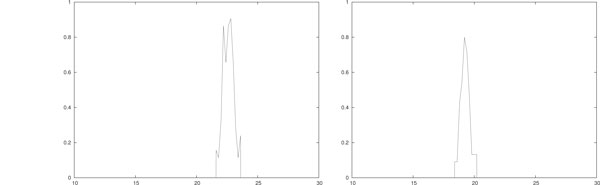
**Probability density function, IL-2 mono-therapy**. Empirical evaluation of *ϱ*(*t_er_*) for different piece-wise constant (left) and impulsive (right) daily IL-2 immunotherapies. In (left) *A *= 0.2 and *d_i _*= 4 · 10^7^/*A *for *i *= 1, . . . , 30. In (right) *u_i _*= 4 · 10^7 ^for *i *= 1, . . . , 30. In both cases *T*(0) = 10^5^, *E*(0) = *I*(0) = 0 and *p_E _*= 20 · 0.1245. All the other parameters are as in Table 3. The densities are obtained by performing 10^2 ^simulations for each configuration.

As we already said some of the simulations we performed are time-consuming, especially when the value of *T *or *E *become huge. This happens, for instance, in the scenarios of Figure 6 where the simulation time spans from 1 to 30 hours. However, single-run of stochastic simulations are not much informative and, when possible, any conclusion should be supported by a big number of averaged simulations. To this extent, we performed multiple runs of both the piece-wise constant and the impulsive IL-2 immunotherapies of Figure 1. This was possible since their running time is of the order of some minutes.

We defined the following time-dependent property over a single simulation: we want to evaluate

$$t_{er}=\min\left\{t\leq 70|T\left(t\right)=0\right\}$$

meaning that *t_er _*is the eradication time for tumor cells before 70 days, more than twice the duration of the simulated therapies. We evaluated the empirical *probability density function *of *t_er_*, denoted *ϱ*(*t_er_*), by performing 10^2 ^simulations for both the scenarios in Figure 1. In all simulations we used the same configuration used in such a figure, that is in (left) *A *= 0.2 and *d_i _*= 4 · 10^7^/*A *for *i *= 1, . . . , 30 whereas in (right) *u_i _*= 4 · 10^7 ^for *i *= 1, . . . , 30. In both cases *T*(0) = 10^5^, *E*(0) = *I*(0) = 0 and *p_E _*= 20 · 0.1245. All the other parameters are as in Table 3.

In case of daily delivery of the IL-2 mono-therapy, Figure 6 shows the evaluation of *ϱ*(*t_er_*) for piece-wise constant (left) and impulsive therapies (right). It is remarkable that for all the simulations the eradication is always reached (i.e. 10^2 ^times out of 10^2 ^simulations).

In Table 5 the average of *t_er_*, denoted 〈*t_er_*〉 and the standard deviation *σ *of *t_er _*are evaluated for the piece-wise constant (left) and impulsive (right) IL-2 immunotherapies of Figure 6. By means of the Wilcoxon statistical test, the non-parametric equivalent of the T-test, we compared the observed realizations of *t_er_*. We obtained that the differences in the values of Table 5 are statistically meaningful since *p <*2.2 · 10^-16 ^for the Wilcoxon statistical test.

**Table 5:** Averages and standard deviation, daily IL-2 mono-therapy

〈*t_er_*〉	*σ*	〈*t_er_*〉	*σ*
19.34	0.33	22.71	0.41

Daily delivered IL-2 immunotherapy. Average of *t_er _*and its standard deviation *σ *evaluated for the piece-wise constant (left) and impulsive (right) therapies of Figure 6.

#### Probabilistic analysis of ACI

As for the case of IL-2 mono-therapy the results on ACIs in Figure 2 motivated us to to investigat the relationship between impulsive and piece-wise constant ACIs, as well as the influence of the period *P *between two consecutive therapy sessions. Again, we considered the same time-dependent property over a single simulation, i.e. *t_er _*= min{*t *≤ 70 | *T *(*t*) = 0}. Also in this case 70 days is more than twice the duration of the therapies that we simulated. We evaluated the empirical probability density function *ϱ*(*t_er_*) by performing 10^2 ^simulations for each of a set of parameter configurations. In all simulations we used the initial configuration *T*(0) = 10^5 ^and *E*(0) = *I*(0) = 0.

**Figure 7:**
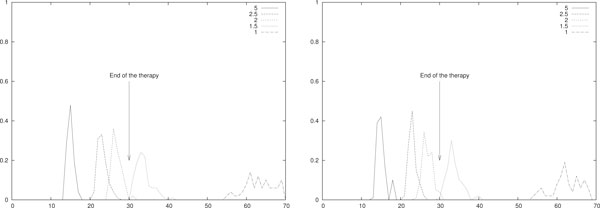
**Probability density function, daily ACI mono-therapy**. Empirical evaluation of *ϱ*(*t_er_*) for different piece-wise constant (left) and impulsive (right) daily ACIs. In (left) *b_i _*= w_* _· 10^4^/(*V A*) for *i *= 1, . . . , 30 and in (right) *w_i _*= w_* _· 10^4 ^for *i *= 1, . . . , 30 where w_* _∈ {5, 2.5, 2, 1.5, 1}. The value of w_* _is given in the figure, all the other parameters are as in Table 3. The densities are obtained by performing 10^2 ^simulations for each value of w_*_.

Figure 7 shows the evaluation of *ϱ*(*t_er_*) for a daily ACIs with piece-wise constant (left) and impulsive (right) infusions. We studied the effect of varying the dosage of the therapy on *t_er_*. Given w_* _∈ {5, 2.5, 2, 1.5, 1}, in (left) each therapy session lasts *A *= 0.2 days and *b_i _*= w_* _· 10^4^/(*V A*) for *i *= 1, . . . , 30 and in (right) *w_i _*= w_* _· 10^4 ^for *i *= 1, . . . , 30. The other parameters are in Table 3.

For any simulation (i.e. 10^2 ^times out of 10^2 ^simulations) the eradication was found if w_* _≠ 1. In the case of w_* _= 1 only half of the simulations predicted eradication before day 70. However, at around day 70 the tumor was small (i.e. always less than 100 cells) meaning that the eradication could have been reached immediately in the days after the 70-th. Interestingly, in both cases it seems that [1.5; 2] is a range for w_* _to have eradication before the end of the therapy, as often desired. In Table 6 the average of *t_er_*, i.e. 〈*t_er_*〉, and its standard deviation *σ *are evaluated for the piece-wise constant (left) and impulsive (right) ACIs of Figure 7.

**Table 6:** Averages and standard deviation, daily ACI mono-therapy

w_*_	〈*t_er_*〉	*σ*	〈*t_er_*〉	*σ*
5	15.5	1.1180	15.2	1.7204
2.5	24.0	2.0	23.5	1.7078
2	27.6	1.9720	27	2
1.5	35.6	3.0397	34.7	3.1638
1	62.0	4.3204	61.0	4.3204

Daily delivered ACI. Average of *t_er _*and its standard deviation *σ *evaluated for the piece-wise constant (left) and impulsive (right) ACIs of Figure 7.

**Figure 8:**
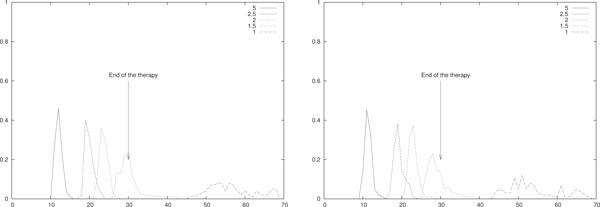
**Probability density function, weekly ACI mono-therapy**. Empirical evaluation of *ϱ*(*t_er_*) for different piece-wise constant (left) and impulsive (right) weekly ACIs. In (left) *b_i _*= w_* _· 7 · 10^4^/(*V A*) for *i *= 1, . . . , 30 and in (right) *w_i _*= w_* _· 7 · 10^4 ^for *i *= 1, . . . , 5 where w_* _∈ {5, 2.5, 2, 1.5, 1}. The value of w_* _is given in the figure, all the other parameters are as in Table 3. The densities are obtained by performing 10^2 ^simulations for each value of w_*_.

In case of weekly delivered therapy, we considered the simulated therapies where the quantity of effectors are injected each week is equal to the one injected per week in the daily therapies above described. This implies *θ_k _*= 1+7 _* _*k*, *k *= 0, . . . , 4 and *b_k _*= w_* _· 10^4^/(*V A*) for piece-wise constant therapy (left) and *w_k _*= w_* _· 10^4 ^(right). The densities are plotted in Figure 8, and the corresponding mean and standard deviation are shown in Table 7.

**Table 7:** Averages and standard deviation, weekly ACI mono-therapy

w_*_	〈*t_er_*〉	*σ*	〈*t_er_*〉	*σ*
5	14.98	0.80	11.39	0.93
2.5	23.12	1.22	19.27	1.30
2	26.81	1.28	22.95	1.23
1.5	33.66	2	28.9	2.42
1	61.18	3.75	53.19	5.70

Weekly delivered ACI. Average of *t_er _*and its standard deviation *σ *evaluated for the piece-wise constant (left) and impulsive (right) ACIs of Figure 8.

By means of the Wilcoxon statistical test we compared the observed realizations of *t_er_*, grouped by kind of delivery (i.e. impulsive or piece-wise constant) and by frequency (i.e. daily or weekly). We obtained that (*i*) if delivered daily the eradication times of the piece-wise constant and the impulsive ACIs are not statistically different (i.e. *p *> 0.05 for all doses). Also, (*ii*) if delivered daily the eradication time of the impulsive therapy is significantly smaller than the one with piece-wise constant therapy (i.e. *p <*10^-12^) and (*iii*) for impulsive ACI the eradication time of the weekly delivered therapy is significantly smaller than the one of the daily delivered therapy (i.e. *p <*10^-12^). Finally, (*iv*) for impulsive ACI the eradication time in the weekly delivery is not statistically different from the one of the daily delivered therapy (i.e. *p *> 0.05).

## Conclusions

In this work we extended our hybrid model [39] with IL-based immunotherapies and Adoptive Cellular Immunotherapies (ACIs), both modeled as piecewise constant or impulsive functions. We performed analytical analysis of the corresponding deterministic model, inspired by earlier work by Panetta and Kirschner [14]. We analyzed our hybrid model via stochastic simulations which seem to suggest results of some interest, which we briefly summarize:

(*i*) by the transitory analysis it turns out that IL-based immunotherapies require very large values of the parameter *p_E_*, which might substantially reduce the number of patients to whom it may be used as monotherapy;

(*ii*) in IL-based immunotherapies the piece-wise constant delivery seems more effective for tumor eradication than the impulsive one although at the price of very long infusion sessions;

(*iii*) in a daily delivered ACI the piece-wise constant delivery seems more or less equivalent to the impulsive one;

(*iv*) in a ACI the impulsive delivery seems slightly more effective than the daily delivery: less frequent deliveries of larger doses ensure a slightly more rapid eradication than frequent deliveries of smaller doses. Note that the latter type of delivery is called metronomic delivery, and it is of great relevance for other anti-tumor therapies such as anti-angiogenesis therapies and chemotherapies [1,59,60]. Furthermore, for those therapies the metronomic delivery is often more effective;

(*v*) in a ACI the weekly impulsive delivery seems slightly more effective than the weekly piecewise constant delivery;

(*vi*) when combined impulsive therapies are considered both the synchronous and the asynchronous delivery seem to be effective and no remarkable differences are observable.

Other more predictable effects were observed such as the synergistic effects of combined therapies, or the dependence of the eradication on the initial values. Of course, these results are strongly linked to the specific model, to its ability in describing the dynamics of real tumors and to the chosen parameters.

As far as the model is concerned, we have previously stressed that maybe the hypothesis that the linear antigenic effect *cT *due to the tumor size should be corrected by assuming a saturating stimulation *cT*/(1+*dT*); here we also add that the assumption that *E*' linearly depend on *E *could be corrected, as there are cases where this dependence might be nonlinear (see [26] and references therein). Note also that, although computationally useful, representing the piece-wise constant delivery of ACI by means of a continuous input *σ_E_*(*t*) is only an approximation. Indeed, in reality the infusion should be more realistically represented as a series of injections of a group of cells each Δ*t *≪ 1 time units. The time interval Δ*t *should be modeled as a Poisson random variable.

As far as the parameters are concerned, in order to obtain more general biological inferences an extensive and systematic exploration of the space of parameters is mandatory. Of course this will require the exploitation of intelligent algorithms (e.g. approximated stochastic simulations [61,62]) to tackle the computational hardness of model analysis.

Finally, here we have only explored the effects of the intrinsic stochasticity on the dynamics of tumor-immune system interplay under therapy. However, it has been shown that without therapy the extrinsic stochasticity may play a significant role in shaping tumor evasion from the immune control [28]. Moreover, it has also been proposed that realistic bounded stochastic fluctuations affecting chemotherapy may deeply influence the outcome of chemotherapies of solid vascularized tumors [63].

Note that the inclusion of realistic extrinsic noise would require minor changes in the proposed hybrid simulation algorithms besides the inclusion of the stochastic nonlinear equations for correlated bounded noises [28,63]. However, that would require extensive numerical simulations (e.g. a higher number of samples of the stochastic process underlying the hybrid system) when inferring heuristic probability densities of eradication times, for instance.

## List of abbreviations used

ACI: Adoptive Cellular Immunotherapy. IL or IL-2: Interleukin-2. ODE: Ordinary Differential Equation. T-IS: Tumor-Immune system (interplay). KP: Kirschner-Panetta (model). SSA: Stochastic Simulation Algorithm.

## Competing interests

The authors declare that they have no competing interests.

## Authors' contributions

AdO conceived of the study and defined the model, GC implemented the model and performed the simulations, AdO, GC and RB analysed the model and wrote the manuscript. All authors read and approved the final version of the manuscript.

## Supplementary Material

Additional file 1 **Supplementary Materials (text)**. Results about scalar differential inequalities, scalar linear ODEs with periodic coefficients, impulsive ODEs and combined impulsive immunotherapies are given. Click here for file
