# Supplementary material for: Fine-tuning anti-tumor immunotherapies via stochastic simulations
Source: BMC Bioinformatics. 2012 Mar 28;13(Suppl 4):S8. doi: 10.1186/1471-2105-13-S4-S8 (PMC3303725; doi:10.1186/1471-2105-13-S4-S8)
Supplement: Additional file 1 — Supplementary Materials (text). Results about scalar differential inequalities, scalar linear ODEs with periodic coefficients, impulsive ODEs and combined impulsive immunotherapies are given. [file 1471-2105-13-S4-S8-S1.pdf]

Fine-tuning anti-tumor immunotherapies via stochastic simulations: Supplementary Materials

Giulio Caravagna[1], Roberto Barbuti[2], Alberto d'Onofrio[3,*]

[1]Institute for Informatics and Telematics, National Research Council, Via. G. Moruzzi 1, Pisa, 56127, Italy.
[2]Dipartimento di Informatica, Università di Pisa, Largo Pontecorvo 3, Pisa, 56127, Italy.
[3]Department of Experimental Oncology, European Institute of Oncology, Via Ripamonti 435, Milano, 20141, Italy.

Email: Giulio Caravagna - giulio.caravagna@iit.cnr.it; Roberto Barbuti - barbuti@di.unipi.it; Alberto d'Onofrio*-alberto.donofrio@ifom-ieo-campus.it;

*Corresponding author

Supplementary Materials

Scalar differential inequalities

Consider the following initial values problem

$$x' = f(x,t), \qquad x(0) = x_0 \tag{1}$$

with $x_0 \geq 0$ and let Q, with $x(0) \in Q$, be a positively invariant set for (1), i.e. such that for all $t > 0$ it is $x(t) \in Q$. Let $g(x,t)$ be a function such that

$$f(x,t) > g(x,t)$$

in Q, and let it be

$$y' = g(y,t), \qquad y(0) = y_0 \tag{2}$$

with $y_0 \geq 0$ and such that Q is positively invariant also for (2). Thus it holds that for all $t > 0$ it is

$$x(t) > y(t)\,.$$

Scalar linear ODE with periodic coefficients

Consider the following linear scalar ODE

$$x' = a(t)x, \tag{3}$$

where $a(t)$ is bounded and periodic with period P. From Fourier's theorem it follows that $a(t) = \langle a(t)\rangle + b(t)$ where $b(t)$ is a bounded periodic function with zero mean. This yelds that

$$x(t) = x(0)\exp\left(\langle a(t)\rangle + \int_0^t b(s)ds\right)$$

and thus, from $x(nP) = x(0)\exp\big(\langle a(t)\rangle Pn\big)$, it follows that $x(t) \to +\infty$ if $\langle a(t)\rangle > 0$, and $x(t) \to 0$ otherwise.

Impulsive differential equations

Consider the following equation

$$x' = -kx + \sum_0^{N_e} q_i \delta(t - \theta_i) \tag{4}$$

where $N_e \leq +\infty$, $k > 0$ and $q_i > 0$ and consider the equivalent linear impulsive differential equation [?,?]

$$x' = -kx, \qquad x(\theta_i^+) = x(\theta_i^-) + q_i \tag{5}$$

since for $\theta_i < t < \theta_{i+1}$ it is $x(t) = x(\theta_i^+)e^{-k(t-\theta_i)}$. We define the auxiliary variable $X_i = x(\theta_i^+)$, whose dynamics is ruled by the following discrete dynamical system

$$X_{i+1} = e^{-k(\theta_{i+1}-\theta_i)}X_i + q_i\,.$$

When $N_e = +\infty$, $\theta_n = nP$ and the doses q_i are all equal, i.e. $q_i = q$ for any i, one has $X_{i+1} = e^{-kP}X_i + q$ and hence

$$X_i = X_\infty + (X_0 - X_\infty)e^{-kPi} \tag{6}$$

where $X_\infty = \dfrac{q}{1-e^{-kP}}$. Then, for large times $x(t)$ tends to

$$x(t) = \frac{qe^{-k(t \bmod P)}}{1-e^{-kP}}\,. \tag{7}$$

Similarly, in the case where k is not a constant but a periodic function $k(t)$ with period P it holds that

$$x(t) = \frac{qe^{-K(t \bmod P)}}{1-e^{-K(P)}} \tag{8}$$

where $K(t) = \int_0^t k(s)ds$.

Combined impulsive immunotherapies

Here we shall briefly study the local stability of a combination therapy between ACI impulsive and IL impulsive. Uniquely to simplify the calculations we consider the special case of contemporary administration, i.e. synchronous therapies where for any i

$$\theta_i^{ACI} = \theta_i^{IL} = iP\,.$$

When all the therapy sessions share the same injection rate of the drug, i.e. $w_i = w$, after some calculations one can show that

$$\varepsilon_\infty(t) = \frac{wF(t \bmod P)}{1-e^{-\mu_E P}\left(\dfrac{Q+g_E}{Qe^{-\mu_I P}+g_E}\right)^{\frac{p_E}{\mu_I}}}$$

where

$$F(t) = e^{-\mu_E t}\left(\frac{Q+g_E}{Qe^{-\mu_I t}+g_E}\right)^{\frac{p_E}{\mu_I}} \qquad Q = \frac{u}{1-e^{-\mu_I P}}\,.$$

The average value of $\varepsilon_\infty(t)$ reads as

$$\langle\varepsilon_\infty\rangle = \frac{w(1+g_E/Q)^{p_E/\mu_I}}{1-e^{-\mu_E P}\left(\frac{Q+g_E}{Qe^{-\mu_I P}+g_E}\right)^{\frac{p_E}{\mu_I}}}\frac{\xi_1}{\mu_E P}$$

where

$$\xi_1 = \int_{e^{-\mu_E P}}^{1} dz\left(z^{\mu_I/\mu_E}+\frac{g_E}{Q}\right)^{-p_E/\mu_I} \qquad z = e^{-\mu_E t}\,.$$

Although very cumbersome, the above integral is analytically calculable since

$$\int\frac{dz}{(z^m+g)^r} = \frac{(z^m+g)^{1-r}}{g}Hyp_{2,1}(\gamma_1,\gamma_2,\gamma_3,\gamma_4)$$

where $\gamma_1 = 1$, $\gamma_2 = 1-r+\dfrac{1}{m}$, $\gamma_3 = 1+\dfrac{1}{m}$, $\gamma_4 = \dfrac{-z^m}{g}$ and $Hyp_{2,1}$ is the Gauss hypergeometric function ${}_pF_q$ of order $(p,q) = (2,1)$ [**?**].
